# The Biology of Ewing Sarcoma

**DOI:** 10.1155/2013/759725

**Published:** 2013-01-10

**Authors:** Keir A. Ross, Niall A. Smyth, Christopher D. Murawski, John G. Kennedy

**Affiliations:** Hospital for Special Surgery, 523 East 72nd Street, Suite 507, New York, NY 10021, USA

## Abstract

*Objective*. The goal of this study was to review the current literature on the biology of Ewing's sarcoma, including current treatments and the means by which an understanding of biological mechanisms could impact future treatments. *Methods*. A search of PubMed and The Cochrane Collaboration was performed. Both preclinical and clinical evidence was considered, but specific case reports were not. Primary research articles and reviews were analyzed with an emphasis on recent publications. *Results*. Ewing sarcoma is associated with specific chromosomal translocations and the resulting transcripts/proteins. Knowledge of the biology of Ewing sarcoma has been growing but has yet to significantly impact or produce new treatments. Localized cases have seen improvements in survival rates, but the same cannot be said of metastatic and recurrent cases. Standard surgical, radiation, and chemotherapy treatments are reaching their efficacy limits. *Conclusion*. Improving prognosis likely lies in advancing biomarkers and early diagnosis, determining a cell(s) of origin, and developing effective molecular therapeutics and antiangiogenic agents. Preclinical evidence suggests the utility of molecular therapies for Ewing sarcoma. Early clinical results also reveal potential for novel treatments but require further development and evaluation before widespread use can be advocated.

## 1. Introduction

Ewing's sarcoma family tumors (ESFT) include Ewing's sarcoma (ES), peripheral primitive neuroectodermal tumors (PNET), and Askin tumors. These tumors are undifferentiated small blue round cell tumors that mainly appear in bone and less frequently in soft tissues [1, 2]. While these tumors are rare, accounting for less than 10% of all human malignancies, they are of the most aggressive and often occur in the long bones and pelvis where they can quickly metastasize to the bone marrow, lung, and other tissues [3, 4]. ES is the second most common bone cancer, most often occurring in Caucasian children, adolescents, and young adults, and is considered a high-grade malignancy [5–8]. Originally, it was thought that ES was derived from primitive neuroectodermal cells; however, there is much debate over the origin of ES. In this regard, endothelial, mesodermal, epithelial, neural, and mesenchymal cells have all been hypothesized as an origin, but there is substantial research indicating that mesenchymal stem cells (MSC) may be the original progenitor of Ewing tumor proliferation [9], and Ewing tumors most often harbor nonrandom balanced chromosomal translocations of the EWS gene on chromosome 22 and any one of several ETS family genes. The most common case is the translocation with the FLI1 gene on chromosome 11 [1, 10]. However, a reciprocal inversion-insertion-translocation mechanism that results in an EWS-ERG fusion gene has also been described [11]. Since the t(11;22)(q24;12) translocation is most frequent, its protein product has been suggested as a main component of ES malignancies [12]. The chimeric proteins in ES play a key role in pathogenesis [13]. EWS protein is thought to be an RNA-binding protein, and FLI1 is thought to be a DNA-binding transcription factor targeting a variety of genes related to a range of functions including apoptosis and differentiation [14]. Although there are only 1–3 cases of ES/PNET per million people/year, recurrence of the cancer has been shown to have a survival rate of as low as 10% and is associated with an increased risk of chronic health conditions. Treatment of ESFTs generally involves combinations of chemotherapy, surgery, and radiation. But, considering that approximately 30% of cases will suffer from relapse, it is critical that cure rates are improved, and morbidity rates are decreased. There is much preclinical evidence that suggests that a greater understanding of the biology and biochemistry of ES/PNET, particularly the activity and expression of EWS-ETS, could advance efficacy of molecularly targeted therapeutics and future treatment [15]. 

## 2. Methods

Extensive searches of both The Cochrane Collaboration and PubMed were performed. Searches included “Ewing sarcoma,” “Ewing's sarcoma,” “Ewing's sarcoma review,” “Ewing's sarcoma AND pnet,” “Ewing's sarcoma family tumors,” “Ewing's sarcoma AND meta-analysis,” “Ewing's sarcoma treatment,” “Ewing's sarcoma AND surgery,” “Ewing's sarcoma therapy,” “Ewing's sarcoma AND mesenchymal stem cells,” “t(11;22)(q24;q12) chromosomal translocation,” “EWS-ETS,” and “EWS-FLI1.” Specific case reports were not reviewed. Primary research articles and reviews were analyzed with an emphasis on recent publications. 

## 3. Chromosomal Translocations

ESFTs are characterized by translocation of the EWS gene with a member of the ETS family genes. A study by Kovar et al. [16] suggests that Ewing's sarcoma is characterized by EWS fusions with FLI1 in 90–95% of cases, ERG in 5–10% of cases, and that FEV, ETV1, and ETV4 fusions occur in less than 1% of cases. There are several studies reporting that a reciprocal translocation of band q24 on chromosome 11 and band q12 on chromosome 22 leads to an in-frame fusion producing an EWS-FLI1 fusion gene in 85% of cases [3, 4, 10]. While the EWS-ERG fusion has also been well documented, due to its complexity incidences of this specific fusion are low [11]. EWS-ETS fusions can vary in chromosomal breakpoints, and there are studies suggesting that differences in breakpoints may be related to varying severities of prognosis [17]. EWS and ETS family combinations are specific to ES, but combinations of EWS with other genes result in a number of other pathologies. It seems to be the case that the partner gene, rather than the EWS gene, is responsible for specifying the tumor type [1]. The function of the EWS protein is not well understood; however, aberrant protein-protein interactions are primarily attributed to the EWS gene, and it is presumed that it is the N-terminus of the EWS that provides the capacity for induction of ES in human cells [3]. Because the EWS-FLI1 fusion is the most common, it may be the case that its transcript is the origin of ES pathologies, and thus it is the fusion gene that has been studied most thoroughly [12]. Fusion of the 5′ segment of the EWS gene with the 3′ segment of the FLII1 gene produces the EWS-FLI1 fusion protein that alters the expression of numerous target genes described below.

## 4. Fusion Proteins and Targets

EWS-ETS chimeric proteins, namely, the EWS-FLI1 protein, are a topic of extensive study because of their tumor specific expression. It is well documented that the EWS-FLI1 protein has an array of downstream targets that contribute to tumorigenesis [18, 19]. For these reasons, EWS-FLI1 is considered a potential therapeutic target. 

EWS-FLI1 has been well documented as both a transcriptional activator and repressor. The function of EWS as a transcriptional activator and FLI1 as the DNA-binding domain is likely responsible for the combined activation/repression abilities of the chimeric protein. Of course, EWS-ETS proteins still require the aid of additional proteins and general transcriptional machinery to function [20]. EWS-FLI1 tends to upregulate genes involved in proliferation, cell differentiation, and cell survival such as IGF1, NKX2, TOPK, SOX2, and EZH2. On the other hand, EWS-FLI1 tends to suppress genes involved in apoptosis and cell cycle arrest including IGFBP3, p57kip, p21, and TGFB2 [3, 21–35]. Microarray analyses have identified over 1000 EWS-FLI1 regulated genes, of which 80% are downregulated targets [18]. There are also a number of secondary events, such as mutations of p53, which have been correlated with prognosis [36]. EWS-FLI1 does not transform human cells *in vitro*, suggesting that the cooperating mutations or related parallel pathways are essential for pathogenesis [18]. Related pathways include p53, INK4A, IGF-1/IGF-1R, bFGF, CD99, and a list of other tyrosine kinase and Wnt pathways [1, 37]. MicroRNAs (miRNAs) are well described in adult cancers and either block target miRNA expression or cause degradation. miRNA-145 has been described as the most active in ESFTs, but other works have shown that other miRNAs could have comparable modulatory effects [37]. Further study of the above pathways, along with RNA processing factors, may shed light onto the specific molecular therapies that could be effective in treating ES/ESFTs. Because of the complexity of ES, and the fact that many different events and players must take part for pathology to occur, the disease is difficult to treat. A comprehensive atlas is needed to improve prognostics, develop of molecular therapies, and eventually treat outcomes. 

## 5. Origins of Ewing's Sarcoma Family Tumors (ESFT)

There is currently no consensus on the cell of origin in Ewing's sarcoma. Identification of the cell of origin has proved problematic, as cellular environment can cause changes in the expression and differentiation effects of EWS-FLI1. Much of the early evidence has pointed towards primitive neuroectodermal and neural crest cells as the cells of origin [3, 38, 39]. Since both ES and PNET contain the same chromosomal translocation, t(11;22)(q24;q12), this hypothesis seemed reasonable. But EWS-FLI1 was shown to induce neuroectodermal differentiation and upregulate genes associated with neural differentiation, leading to the hypothesis that the neuroectodermal features of ES may simply be the result of EWS-FLI1 expression [3, 18, 40–43]. It is also important to consider that the long bones of the limbs originate from the mesoderm, so in normal conditions, primitive neuroectodermal cells may not even be present in bone. 

In light of the above assertions, MSCs have repeatedly been hypothesized as the cellular progenitor of ES. MSCs are bone marrow derived, making them a feasible candidate for the ES cell of origin considering most cases of ES appear in bone. Murine studies have demonstrated that EWS-FLI1 can transform bone marrow derived MSCs to ES-like tumors and induce MSC transformation in the absence of other oncogenics, and that EWS-FLI1 and EWS-ERG blocked proper differentiation in MSCs [44–46]. In human ES cell lines, it has been shown that EWS-FLI1 silencing results in convergence to a gene expression profile similar to MSCs. The same group was able to demonstrate that ES cell lines could also differentiate along osteogenic lineage when EWS-FLI1 was under long-term inhibition [9]. However, when human MSCs were infected with a retrovirus containing EWS-FLI1, tumors fail to develop in mouse hosts [47]. Therefore, EWS-FLI1 on its own may not cause cell transformation and tumor growth. It may be the case that cell transformation and ES tumor growth only occur in the presence of EWS-FLI1 when specific mutations already exist in a given cell [3]. Because neural-derived MSCs are present in bone marrow, and neural crest cells contain mesenchymal lineage plasticity, Riggi et al. suggested that the concept of neuroectodermal/neural crest and MSCs as the cells of origin need not be mutually exclusive [18, 48]. A recent study has demonstrated that EWS-FLI1 was well tolerated and led to alteration of expression in target genes in both human embryonic stem cell derived neural crest stem cells and neuromesenchymal stem cells [49]. Coming to a consensus on the cell of origin in ES is crucial in developing earlier detection of sarcomagenesis. Knowledge of early precursor cells is also vital to accurately describing pathogenesis and finding molecular therapies that provide more target specific clinical treatments. 

## 6. Clinical Approaches and Targeted Therapeutics

Localized pain is the most common early symptom of ESFTs. Since many ES patients are young and are often physically active, the pain is frequently mistaken for bone growth or injury. This is clearly an issue as it can result in a delayed or misdiagnosis [50, 51]. ES often progresses rapidly and results in visible or palpable swelling, but tumor bulk and swelling can be difficult to detect in femoral, pelvic, chest wall, or spinal tumors. Diagnostic imaging initially includes a two plane radiograph, followed by magnetic resonance imaging (MRI) to more accurately define the local extent of a given tumor as well as the relation to nearby blood vessels and nerves. MRI and/or computed tomography (CT) are currently the imaging standard, but there are recent studies suggesting that PET, thallium-201 scintigraphy, and dynamic MRI may provide more valuable information [52–55]. If a biopsy is to be taken following imaging, it is recommended that the surgeon perform it, so that the incision is in an appropriate location with consideration to a definitive surgery, especially when limb salvage will be attempted [56]. Advances in diagnosis will continue to play a key role in improving cure/survival rates of ESFT.

### 6.1. Standard Treatment Options

Due to the high lethality of ESFTs, much of the literature advocates an aggressive multidisciplinary approach. This approach typically refers to a combination of surgery, chemotherapy, and radiation. All three forms of treatments have shown efficacy in some cases [52]. Most current therapies call for multidrug chemotherapy, consisting of cycles of varied combinations of vincristine, doxorubicin, cyclophosphamide, etoposide, ifosfamide, actinomycin D, and topotecan, followed by local therapies (radiation and/or surgery) [8, 53]. 

### 6.2. Local Control/Local Tumors

Radiotherapy was regarded as the standard treatment for local/focalized tumor therapy for decades [53]. However, the current evidence advocates surgery when complete resection is possible [57, 58]. Resection, with preoperative and postoperative chemotherapy, without postoperative radiation is the treatment of choice when large margins around the tumor can be achieved. Several groups have shown that this approach is preferable when surgery is possible, and that limb salvage procedures can be performed without a decrease in survival rates [57–61]. Surgical resection margins are most often defined as described in the Ennecking surgical staging of osteosarcoma [60]. At this point in time, there are no randomized control trials comparing local control therapies (radiotherapy versus surgery). For this reason the preferred local treatment for ES is a topic of debate. Radiation therapy, as the only form of therapy, is typically the treatment of choice for patients with large tumors or with tumors in locations that make surgical resection not possible. It is important to consider that studies on radiotherapy might be comprised of an unfavorable subject group [52, 53]. A study by Schuck et al. reviewing 1058 cases showed that patients who received resection of a portion of the tumor, often referred to as “debulking,” with subsequent radiotherapy presented local control rates that did not differ from those receiving radiotherapy alone [57]. Due to the above evidence, or lack thereof, it seems that until further development, the choice of local control therapy should be made on a case-by-case basis [53]. With the current developments in treatment modalities, the survival rate of patients with localized ES approaches 70% five years after diagnosis [62, 63]. 

### 6.3. Metastatic Tumors

The prognosis of patients with metastatic tumors drops significantly compared to localized tumor cases [64]. In 25% of ES cases, there is evidence of metastatic disease, which is regarded as the most adverse prognostic factor [53, 62–65]. Patients with combined bone, bone marrow, and lung metastases have been shown to have a 4-year event free survival (EFS) rate of as low as 14% [64]. Treatment regimens include chemotherapy in combination with radiation therapy. Intense therapies that include higher doses of chemotherapy, sometimes along with total body radiation and stem cell support, have not shown increased EFS rates [66, 67]. Improving cure rates and increasing EFS rates have proved to be a challenge in patients presenting with metastatic disease, and potential molecular therapies are likely the means through which the prognosis may be improved.

### 6.4. Recurrent Tumors

30–40% of patients suffer from recurrent tumors either locally, distally, or some combination of the two, and their prognosis remains poor [68]. Upon recurrence, chances of survival have been estimated at less that 20–25% [69]. Treatment for recurrent cases also typically involves some combination of chemotherapy, surgery, and radiation therapy, with treatment types and doses based on extent of local recurrence and presence of metastatic disease upon recurrence. There is no specific treatment regimen established for recurrent cases, primarily because of the variance between cases. Treatment should be altered according to the individual treatment provided to the patient before recurrence. Again, molecular therapies seem to be the means through which prognosis and ESF rates can be improved. Because of the poor prognosis associated with recurrence and because patients with metastatic presentation are more likely to suffer from recurrence, these therapies should be focused on halting metastases and recurrent tumor formation [65]. 

### 6.5. Molecular Therapeutic Targets

Some of the most promising therapeutics have been outlined by Kelleher and Thomas which include EWS-FLI1 gene silencing, IGF-IR1 antagonists, mTOr inhibition, KIT oncoprotein targeting, CD-99-directed monoclonal antibody treatment, allogenic NK cell immunotherapy, tumor necrosis factor-related apoptosis, histone deacetylase inhibitors, NKX2 transcriptional repression, along with a list of other kinases inhibitors [15]. Other potential strategies include the use of small interfering RNAs (siRNA), YK-4-279 to block RNA helicase A binding, O-linked beta-N-acetylglucosamine, miRNA (specifically miRNA-145) targeting, and various antiangiogenic and antivascular strategies [15, 37, 70]. 

 Therapeutics based on antisense cDNA and siRNA have been shown to reduce EWS-FLI1 expression and increase survival rates in mice with ESFTs present [71–73]. However, due to the fact that these types of therapeutics are challenging to administer pharmacologically, they are not necessarily applicable clinically, and several groups have asserted that the next reasonable step is focusing on protein-protein interactions rather than the ESFT genes themselves [15, 37, 74]. In contrast, insulin-like growth factor receptor-1 (IGF-1R) antagonists include a list of antibodies and tyrosine kinase inhibitors, which have shown responses in phase I and phase II clinical trials. A phase I study performed in 2010 treated a group of patients with the monoclonal antibody targeting IGF-1R-figitumumab. 16 of these patients had Ewing's sarcoma, one of which had a partial response, another had complete response, and 6 patients with ES had disease stabilization that lasted from 4 to 16 months [75]. In another phase I/II trial of figitumumab, 16 patients with ES showed no dose limiting toxicity, and during the subsequent phase II trial, 106 patients were available with 25 showing stable disease and 15 showing partial response [15, 76]. The R1507 monoclonal antibody has also been studied in phase II clinical trials. In 125 patients with ES/PNET treated with R1507, 18 patients had a complete or partial response [77]. In addition, a phase II study by the same group showed a complete or partial response rate of 10% in a group of 133 patients [77]. It has also been suggested that combining IGF-1R inhibitors with mTOR inhibitors may improve efficacy [15, 37]. A 2011 study phase I trial revealed that this combination could be well tolerated, and tumor reduction was seen in 2 of 3 patients with ES and 4 of 10 patients with adrenocortical carcinoma [78]. The review by Kelleher and Thomas, published in 2012, compiled phases I and II data and concluded that the response rate to IGF-R1 therapeutics was about 10% [15]. However, there was a phase II study of a novel mTOR inhibitor that reported a 30% clinical benefit rate in 50 patients with bone sarcoma [79].

The Children's Oncology Group investigated a different target site by examining KIT oncoprotein inhibition in a phase II study by treating 70 patients with imatinib mesylate. 24 of these patients had ES/PNET, and only one partial response was observed. No responses were observed in the other cancer patients [80]. Few clinical studies have been performed to specifically investigate antiangiogenic and antivascular effects of molecular strategies in ES. Bevacizumab is a US Food and Drug Administration approved antiangiogenic agent that has been used in a clinical setting. A phase I study including 5 ES patients resulted in stable disease for one patient for 4 months, and two patients had stable disease for 9 months while being treated with bevacizumab every 2 weeks [81]. Four ES patients treated with bevacizumab and liposomal doxorubicin in another study resulted in two patients with stable disease for 10 months [82]. The National Cancer Institute and Children's Oncology Group have been conducting clinical trials of ES-targeted molecular therapies, including IGF inhibitors, mTOR inhibitors, and NK cell and other immunotherapies, but most have been phases I and II trials. Clinical evaluation of ESFT molecular therapies is in the early stages and will require more study and phase III randomized trials with larger subject groups before further development occurs.

## 7. Conclusions

ES is a rare pathology; however, it is a highly lethal disease that has a poor prognosis when metastatic or following recurrence. In contrast to localized cases of ES, advances in standard treatment approaches have not been encouraging with regard to improving the survival rates for metastatic and recurrent cases, as 5-year survival rates have recently been described as less than 25% [8, 62, 63]. It has been suggested that chemotherapy and radiation treatments are reaching their efficacy and toxicity limits [83]. In light of these findings, it becomes increasingly essential to improve the efficacy and utilization of targeted molecular therapies. Although it will clearly be difficult, there is much opportunity available to improve biomarkers used to identify targets as well as therapies for biological/molecular treatment of such targets. A critical gap in the research is finding a consensus on the cell of origin of ESFTs, which could help to make earlier detection of ES possible. The understanding of the chromosomal expression of ESFTs and their transcripts has grown substantially, but more knowledge is needed to advance therapeutics. Targeting protein-protein interactions, especially of EWS-FLI1, seems promising, and antiangiogenic therapies appear to have potential for combating metastatic and recurrent cases as well. Further study of the biology of the disease and consequent development of targeted therapies will likely be the means to improve prognosis and survival rates of ES patients.
